# Ifosfamide May Be Safely Used in Patients with End Stage Renal Disease on Hemodialysis

**DOI:** 10.1155/2009/575629

**Published:** 2010-01-04

**Authors:** Sheron Latcha, Robert G. Maki, Gary K. Schwartz, Carlos D. Flombaum

**Affiliations:** Memorial Sloan Kettering Cancer Center, 1275 York Avenue, NY 10065, USA

## Abstract

*Background*. Pharmacokinetic data on clearance of ifosfamide in hemodialysis patients are limited. Consequently, these patients are excluded from therapy with this agent. We review the outcomes for patients at our institution with end stage renal disease on dialysis who received ifosfamide for metastatic sarcoma. *Patients and Methods*. We treated three patients with end stage renal disease on hemodialysis with escalating doses of ifosfamide. Data on radiographic response to therapy, WBC and platelet counts, signs or symptoms of infection, neuropathy and bladder toxicity are reported. Starting doses of ifosfamide were based on review of the literature available with subsequent modifications based on each patient's prior exposure to myelosuppressive agents and on symptoms of neurotoxicity and the degree of myelosuppression following each cycle of chemotherapy. *Results*. Myelosuppression was the most common side effect from therapy, but no patient developed a life threatening infection, neurotoxicity, or hematuria. One patient developed epistaxis in the setting of thrombocytopenia while on warfarin therapy. All patients had clinical evidence for therapeutic response and two had documented radiographic improvement following ifosfamide administration. *Conclusion*. Ifosfamide can be used safely in combination with hemodialysis in patients with end stage renal disease.

## 1. Introduction

Ifosfamide has been shown to consistently achieve response rates of at least 25% when used as a single agent in patients with several types of recurrent and refractory sarcomas [1, 2]. A member of the oxazaphosphorine family of alkylating agents, ifosfamide is primarily metabolized by the liver [3]. The prodrug is transformed by cytochromes to an active phosphoramide mustard, but also generates the urotoxin acrolein, and the neurotoxin and nephrotoxin, chloracetaldehyde. Although renal clearance in patients with normal renal function does not usually exceed 20 ml/min [4–6], and no important pharmacokinetic changes should appear in patients with renal impairment, an accumulation of toxic metabolites has been observed in patients with underlying renal insufficiency [7–10]. Renal excretion of some of the nontoxic metabolites of ifosfamide, 2 and 3-dechloroethyl ifosfamide, has been shown to account for between 4–13% of the administered ifosfamide dose [7]. Chloracetaldehyde, a metabolite implicated in CNS toxicity, has been shown to accumulate in patients with renal insufficiency [9, 11] and in an anephric patient on hemodialysis [12]. In vitro studies suggest that hemodialysis can decrease ifosfamide concentrations by 87% and chloracetaldehyde by 77% [13]. In a report of a patient who mistakenly received a rapid infusion of 9 g of ifosfamide over 1 hour, combined modality therapy with hemodialysis and hemoperfusion decreased serum concentrations of ifosfamide by 84% and 9%, respectively [14]. To date, there are only one case report in the world's literature on the use of ifosfamide in an anephric patient on hemodialysis [12] and one case report on the use of hemodialysis to treat ifosfamide toxicity [14]. We present our experience with three patients with end state renal disease on hemodialysis who were treated with reduced doses of ifosfamide, which were increased as tolerated.

## 2. Materials and Methods

After obtaining approval from our institutional review board, we retrospectively reviewed the medical records of three patients as outlined below.

## 3. Results

**Figure 1:**
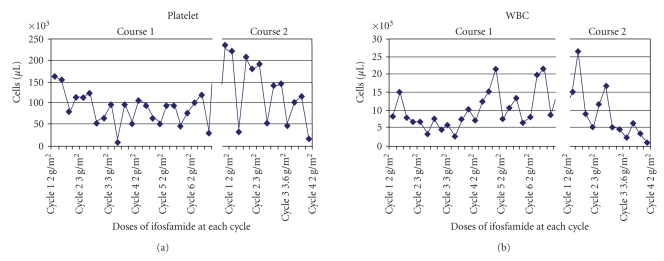
Dose of ifosfamide with nadir platelet and white blood cell counts.

Table 1 summarizes the dosing schedule and toxicity associated with ifosfamide administration with each of the three cases. A graph of the WBC and platelet counts in response to varying doses of ifosfamide used in Case 1 is represented in Figure 1.

### 3.1. Case Histories

**Case 1**

Case 1 was a 48-year-old male who four years earlier had been diagnosed with high grade synovial sarcoma of the knee, metastatic to lungs. The patient was referred to the renal service, while on hemodialysis, for consideration of the feasibility of ifosfamide chemotherapy in a patient with end stage renal disease. In the past, whenever chemotherapy with this agent had been stopped, his disease recurred rapidly. An 18-month course of ifosfamide chemotherapy became complicated by ifosfamide nephrotoxicity and the progression to end stage renal disease, necessitating the initiation of hemodialysis. Based on a previous report describing the use of ifosfamide administration in a patient on hemodialysis (12), we decided to institute treatment with ifosfamide at reduced doses and increased the dose as tolerated.

Chemotherapy was delivered as outlined in Table 1, Case 1. Hemodialysis was delivered within 10–14 hours after the initiation of ifosfamide administration. Treatment was initiated at a dose of 2 g/m^2^, and was then increased to 3 g/m^2^ for the next 2 cycles. Each dose was divided evenly over two consecutive days. Thrombocytopenia, with a nadir platelet count of 5 K/ul (NCI grade 4), was observed after the 3rd cycle, prompting a return to 2 g/m^2^ for all ensuing cycles during that initial 6-month treatment period. Subsequently, there was a 14-month period during which the patient did not receive ifosfamide during which new lung lesions appeared and he underwent 3 additional lung resections in a period of 6 months.

For Course 2, ifosfamide was started at a dose of 2 g/m^2^, and was then increased to 3 g/m^2^, 3.6 g/m^2^, and finally 4 g/m^2^, given as split doses over 2 consecutive days. After the 4 g/m^2^ dose, he developed epistaxis associated with a platelet count of 17 K/uL (NCI grade 1), while on warfarin therapy for a DVT. The details of the platelet and WBC counts with each cycle over both courses are represented in Figure 1. The patient expired from progression of disease 3 months later, 7 years after the initial diagnosis of metastatic synovial sarcoma.

**Case 2**

This is a 51-year old Asian female with a history of metastatic leiomyosarcoma, who, after developing a rash on paclitaxel, opted to be treated with Chinese herbal medications. In the setting of ureteral obstruction, her renal insufficiency, which was believed due at least in part to Chinese herbal nephropathy, progressed to dialysis dependent end stage kidney disease. Following disease progression despite doxorubicin, the decision was made to treat with ifosfamide. Chemotherapy was delivered as outlined in Table 1, Case 2. Each cycle of ifosfamide was given as one dose, three weeks apart. Escalating doses of ifosfamide were administered over three cycles as follows: 1.5 g/m^2^, 1.8 g/m^2^, and 2 g/m^2^. The first cycle was administered as an inpatient and was followed by hemodialysis approximately 3 hours after ifosfamide infusion. Cycles 2-3 were given as an inpatient and the patient then received her regular hemodialysis at her local outpatient dialysis unit 2-3 hours later.

The patient did report some softening of her intraabdominal masses and decreased abdominal distension following the first two cycles. A repeat scan done after cycle 2 showed no interval increase in tumor size. Two weeks after the final cycle, she reported painful neuropathy in the left lower extremity. A consulting neurologist felt that this presentation and distribution of symptoms was more consistent with a plexopathy as opposed to a toxin mediated neuropathy. No spinal involvement with tumor was noted on MRI or CT of the abdomen and pelvis. Imaging performed after the third cycle showed interval decrease in the size of some intraabdominal nodules with additional nodes in the retroperitoneum and peritoneal cavity and new hydronephrosis and hydroureter. Due to chronic pain and progressive disease, the patient opted for palliative care. She expired 8 months later.

**Case 3**

In December 2007, a 67-year-old male presented with progressive metastatic myxoid/round cell liposarcoma of the right calf, associated with increased calf pain and difficulty with ambulation. He had received neoadjuvant therapy with ifosfamide and doxorubicin and underwent surgical resection of the lesion when the tumor size decreased. He progressed to end stage kidney disease and the decision was made to treat with ifosfamide since he had a response to this agent in the past. Chemotherapy was delivered every 3 weeks as outlined in Table 1, Case 3. Dialysis was scheduled within 10 hours after the end of each dose of ifosfamide. The patient received a starting dose of 1.5 g/m^2^, which was increased 3 weeks later to 1.8 g/m^2^. Both doses were split evenly over 2 days. Following both cycles, he developed cellulitis in the affected leg, NCI grade 2. After Cycle 1, the patient left the hospital walking and his pain was well controlled. In light of recurrent cellulitis following both cycles of ifosfamide, the patient opted for chronic suppressive therapy with oral antibiotics and treatment with ifosfamide was terminated. Unfortunately, there was no repeat imaging to document response to therapy, but the treating physician believed he had clinical evidence of disease progression. While on ifosfamide therapy, the patient continued to require PRBC transfusions.

All three patients were nonoliguric. Therefore, furosemide 200 mg IV was given over one hour prior to the infusion of ifosfamide in an attempt to increase urine flow and minimize the possibility of hemorrhagic cystitis. Mesna was given IV every 4 hours at 1/3 the ifosfamide dose, with the first dose 1/2 hour prior to chemotherapy. In order to avoid overhydration, IV fluids as 5% dextrose in 0.45% saline was given at a rate equal to the urine output. For patients 1 and 3, to coordinate the chemotherapy administration with dialysis, patients were hospitalized in the afternoon and ifosfamide was administered in the evening. Other medications administered at the time of chemotherapy included antiemetics (granisetron, dexamethasone ,and metoclopramide) as well as granulocyte colony stimulating factor, given 1-2 days after completion of chemotherapy.

All three patients initially showed clinical response following therapy with ifosfamide, though the benefit was brief in patient 3. There was radiographic evidence showing response to treatment and lack of disease progression in patients 1 and 2, respectively.

Myelosuppression was the most common side effect from ifosfamide treatment. Over a course of 15 total cycles, 1 cycle was associated with grade 4 neutropenia and 4 cycles with grade 3 thrombocytopenia. With regard to neurologic sequelae, no patient developed seizures, sedation, tremors, or irritability. Patient 2 developed neuropathic pain confined to the left lower extremity. As per a neurologist's assessment, based on the distribution of the pain, timing after exposure to the alkylating agent, and MRI results, it was concluded that the pain was most consistent with a plexopathy rather than a toxin mediated neuropathy. Patient 3 had NCI grade 2 cellulitis. No patient developed hemorrhagic cystitis.

## 4. Discussion

In formulating our approach for establishing the starting dose of ifosfamide and the timing of hemodialysis following ifosfamide infusion, we relied heavily on the pharmacokinetic and toxicity data on an anephric pediatric patient on dialysis who was treated with ifosfamide for a Wilms' tumor [12]. For this pediatric patient, in course 1, the patient was given one dose of 1.6 g/m^2^ of ifosfamide followed by dialysis at 24 hours. Significant neurotoxicity, including seizures, tremors, and irritability occurred. In course 2, a dose of 1.6 g/m^2^ was followed 72 hours later by 1 g/m^2^. Dialysis was started 7 hours after each dose. This regimen produced less neurotoxicity but more leukopenia and thrombocytopenia, with nadir ANC and platelet counts of 440/uL and 23 K/uL, respectively. In course 3, 1 g/m^2^ was given every 48 hours for 3 doses and dialysis was started at 7 hours from end of dose. This produced neurologic toxicity comparable to course 2 with less neutropenia and thrombocytopenia. The nadir ANC and platelet counts were 1.2 K/uL and 59 K/uL, respectively. In the last course, the patient was given four daily doses of 1 g/m^2^, again followed by dialysis at 7 hours post treatment. Decreasing the interval between dosing and increased frequency of dosing produced more neurologic toxicity and myelosuppression. The nadir ANC and platelet counts were 414/uL and 5 K/uL, respectively. 

In light of this data, in Case 1, we felt that it would be best to begin ifosfamide therapy with approximately 2 g/m^2^ of, divided over two consecutive days, and to then titrate the dose and frequency of ifosfamide administration based on clinical and laboratory parameters. A priori, we did not know if this was an optimal dose for treatment of a synovial sarcoma but, based on the pediatric report, wished to avoid significant neurotoxicity and myelosuppression. Hemodialysis was performed 10–14 hours after ifosfamide administration in order to allow for enough exposure to the drug. Thereafter, the starting dose was adjusted based on signs and symptoms suggesting neurotoxicity and the degree and duration of myelosuppression following each cycle of chemotherapy. 

In Case 2, we referred back to the data on the anephric patient and administered as a single dose of 1.5 g/m^2^. The referenced anephric patient experienced significant neurotoxicity when given a single dose of 1.6 gm/m^2^ followed by dialysis at 24 hours. To offset the neurotoxicity observed in course 1 with the anephric patient, and for logistical reasons, we opted to dialyze at 3 hours instead of at 24 hours. This decision had been based on data that showed that chloracetaldehyde, the most likely neurotoxic metabolite of ifosfamide, had peak levels at 4 hours following ifosfamide infusion and that chloracetaldehyde levels decreased by a mean of 77.2% following dialysis [12]. Since our patient received dialysis at a nearby outpatient facility, at her request, dialysis was performed at her outpatient unit within 3 hours of ifosfamide infusion for cycles 2 and 3. 

Case 3 had underlying myelodysplastic syndrome. In light of this, we decided on a lower starting dose, 1.5 g/m^2^, divided over 2 consecutive days, followed by dialysis within 10 hours of chemotherapy. The patient had a brief initial clinical response to this dosing schedule with no significant myelosuppression but developed cellulitis, and clinical disease progression, precluding further dose increments. Ifosfamide was not administered at consistent intervals in all cases and, with the exception of patient 1, the number of cycles was limited.

Based on the nadir WBC and platelet counts achieved, it appears that only Case 1 achieved a myelosuppressive dose. Indeed, this is the only patient who experienced long term benefit from ifosfamide therapy. Unfortunately, data on serum concentrations of ifosfamide and its metabolites were not collected at the time of drug administration and, because of the retrospective nature of this report, we are unable to provide pharmacokinetic data for these 3 patients on hemodialysis. None of the patients had the same tissue tumor type, and it is possible that chemosensitivity of the different soft tissue sarcoma tumor types may have an impact on tumor response. Only modest doses of ifosfamide were used in these 3 cases, raising the possibility that higher doses may have had a different effect in Cases 2 and 3 since neither patient received myelosuppressive doses.

Although all three patients were dialysis dependent, they did have some residual urine output and may have potentially been at risk for hemorrhagic cystitis from the effects of acrolein accumulation within the bladder. Although there are no data regarding the urinary excretion of this metabolite in patients with dialysis dependent renal failure, it is possible that the risk of hemorrhagic cystitis may be negligible if the urotoxic metabolites are not excreted, because of the low glomerular filtration rate and resultant diminished urine output. If this were the case, then hydration prior to ifosfamide may not be necessary in patients who are dialysis dependent. Since all were at risk for volume overload, congestive heart failure, and hypertension if exposed to the usual pretreatment hydration for ifosfamide administration, we used a modified hydration protocol to mitigate bladder toxicity and volume overload, outlined in Table 2. No patient experienced hemorrhagic cystis or volume overload on this protocol. 

Ifosfamide, alone or in combination with other chemotherapeutic agents, plays a significant role in the treatment of solid tumors. As illustrated in the first case scenario, this may be the only drug to which a patient achieves sustained control of their metastatic disease. In Case 1, the patient had 7 years of sustained response to ifosfamide, during which time he continued to work and to take frequent cruises. The retrospective nature of this report; the lack of uniformity in the dose of ifosfamide and the interval to dialysis and the absence of pharmacologic data are all major limitations of this case report. However, given the paucity of pharmacokinetic data on renal and dialysis clearance of ifosfamide, and the exclusion of patients with significant renal insufficiency in clinical trials, our reported experience does provide clinically meaningful information for the oncologist caring for patients with severe renal impairment. 

Pharmacokinetic data on renal clearance and dialysis clearance of ifosfamide are limited. However, the existing data and the present case series support the use of this alkylating agent in patients with end stage renal disease on hemodialysis. Judicious dosing based on the combined effects of prior myelosuppressive agents and concomitant bone marrow disease, combined with timely administration of hemodialysis and appropriate hydration, can avail patients to the therapeutic effects of this agent while minimizing the neurotoxicity, myelosuppression, and the risk for hemorrhagic cystitis. Moreover, with thoughtful consideration of the dose limiting toxicity of ifosfamide, the administration of this therapy can be modified to accommodate outpatient hemodialysis. 

## Figures and Tables

**Table 1:** Dosing schedule, nadir counts and reported complications in Cases 1, 2 and 3.

Case 1				

Course 1				

	Ifosfamide dose/m^2^*	Nadir WBC (K/uL) [ANC (K/uL)]	Nadir PLT (K/uL)	Complication

Cycle 1	2 g/m^2^	6.7 [4.7]	81	
Cycle 2	3 g/m^2^	3 [2.9]	53	
Cycle 3	3 g/m^2^	2.6 [2.3]	5	Thrombocytopenia NCI grade 4
Cycle 4	2 g/m^2^	7 [4.5]	50	
Cycle 5	2 g/m^2^	6.4 [5.4]	44	
Cycle 6	2 g/m^2^	7 [5.3]	30	

14-Month interval				

Course 2				

Cycle 1	2 g/m^2^	8.9 [6.8]	35	
Cycle 2	3 g/m^2^	5 [4.6]	55	
Cycle 3	3.6 g/m^2^	2.2 [1.3]	48	
Cycle 4	4 g/m^2^	0.79 [0.26]	17	Epistaxis NCI grade 1

Case 2				

Cycle 1	1.5 g/m^2^	4.3 [3.3]	125	
Cycle 2	1.8 g/m^2^	3.8 [2.7]	115	
Cycle 3	2 g/m^2^	8 [7.4]	95	

Case 3				

Cycle 1	1.5 g/m^2^	8.9 [6.3]	106	Cellulitis NCI grade 2
Cycle 2	1.8 g/m^2^	13.7 [8.4]	334	Cellulitis NCI grade 2

*Note: For Cases 1 and 3, this represents the total dose of ifosfamide, split over 2 consecutive days

Case 2 received a single dose of ifosfamide per cycle.

**Table 2:** Proposed plan for nonoliguric patients with ESRD on dialysis receiving ifosfamide.

Day 1	Admit patient in the afternoon
	Forced diuresis:
	Furosemide 200 mg IVP over 1 hour
	Match urine output with D5 + 0.45%NS
	*(if patient oliguric/anuric, furosemide, Mesna, and IV fluids are not needed) *
	Ifosfamide 1-2 g/m^2^ + Mesna, starting ifosfamide at 4–6 PM
Day 2	HD in the AM (10–12 hrs after chemotherapy)
	Repeat furosemide and IV fluids as above
	Ifosfamide + Mesna, starting ifosfamide at 4–6 PM
